# Supplementary figures and images for: A unifying structural and functional model of the coronavirus replication organelle: Tracking down RNA synthesis
Source: PLoS Biol. 2020 Jun 8;18(6):e3000715. doi: 10.1371/journal.pbio.3000715 (PMC7302735; doi:10.1371/journal.pbio.3000715)

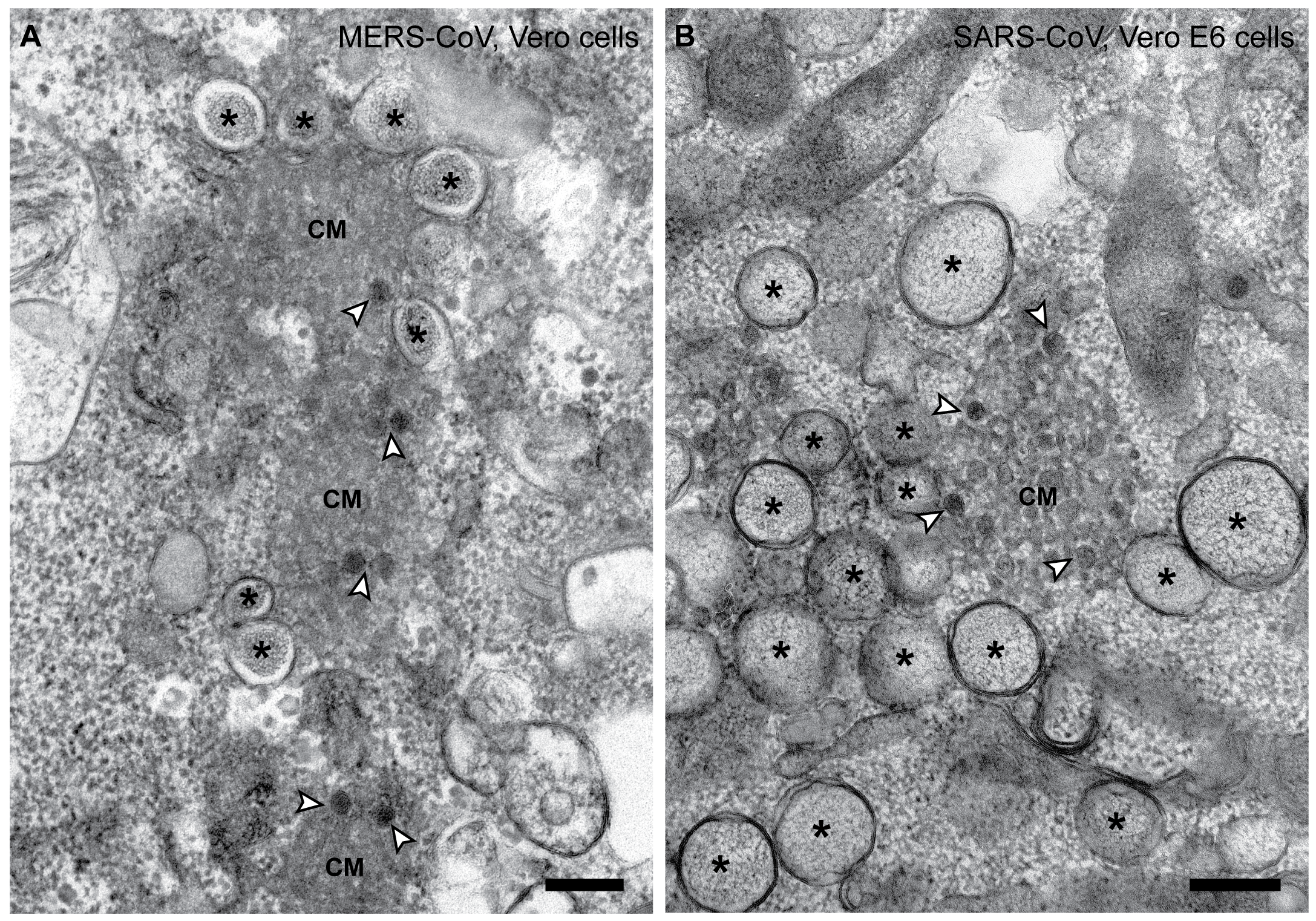

Supplement: S1 Fig — Analysis of previously described samples of CoV-infected cells, prepared for EM either by HPF (A) or cryo-plunging (B). A targeted search revealed the presence of DMSs (white arrowheads) in close association with CM. In comparison with the chemically fixed samples used in this study, the superior ultrastructural preservation of cryo-fixation results in less distorted membranes, but also in a denser cytoplasm and darker CM that makes DMS less apparent. (A) Example from a MERS-CoV-infected Vero cell (16 hpi) in a sample used in [21]. (B) Region in a SARS-CoV-infected Vero E6 cell (8 hpi), adapted from [15]. Scale bars, 250 nm. CM, convoluted membranes; CoV, coronavirus; DMS, double-membrane spherule; EM, electron microscopy; FS, freeze-substituted; HPF, high-pressure freezing; hpi, hours postinfection; MERS-CoV, Middle East respiratory syndrome-coronavirus; SARS-CoV, severe acute respiratory syndrome-CoV. (TIF) [file pbio.3000715.s002.tif]
