# Supplementary material for: A unifying structural and functional model of the coronavirus replication organelle: Tracking down RNA synthesis
Source: PLoS Biol. 2020 Jun 8;18(6):e3000715. doi: 10.1371/journal.pbio.3000715 (PMC7302735; doi:10.1371/journal.pbio.3000715)
Supplement: S1 Text — (DOCX) [file pbio.3000715.s003.docx]

# Supporting Information

## S1 Text: Autoradiography in electron microscopy

Autoradiography is a classic technique that allows the EM visualization of a radioactive marker, usually targeting a certain process, and thus reveals the subcellular localization of that process [1-2]. Tritiated uridine, for example, can be used to locate active RNA synthesis [3-5], as also shown in this study. A clear advantage over the use of alternatives for metabolic labelling of newly-synthesized RNA (e.g. Br-uridine, Br-UTP, 5-ethynil uridine) is that the radioactive precursor is chemically identical to the natural substrate.

After labelling, the samples are immediately fixed and processed for EM. The location of the radioactive marker can then be made apparent by applying a highly-sensitive photographic emulsion (a nuclear emulsion) on top of the cell sections and exposing it for several weeks to months. The beta particles that are emitted as a result of tritium disintegrations generate electrons that get trapped in the silver halide emulsion and create a “latent image”. When the emulsion is developed, these negative charges promote the reduction to metallic silver, generating electron-dense grains that are visible by EM. In principle, given enough time to accumulate enough radioactive disintegrations, even low levels of the radioactive marker could be detected. In practice, other factors (e.g. background radiation, emulsion aging) set some limits to autoradiography, which is nonetheless a very sensitive technique.

The resolution of EM autoradiography is limited by the fact that radioactive disintegrations generate beta particles that are emitted in random directions. Importantly, the probability of giving rise to signal decreases with the distance from the radioactive source; however, some beta particles may travel up to a few hundred nanometers before striking the photographic emulsion [2]. Therefore, it is important to keep in mind that the silver grains may not directly overlay the structure containing the radioactive source. Quantitative analyses of the signal that take this factor into account, like those presented in this study, become indispensable to maximize the information that autoradiography can provide.

## References

1. Bienz KA. Techniques and applications of autoradiography in the light and electron microscope. Microsc Acta. 1977;79(1):1-22. Epub 1977/01/01. PubMed PMID: 65723.

2. Bozzola JJ, Russell LD. Autoradiography & Radioautography. Electron Microscopy: Principles and Techniques for Biologists. Sudbury, MA.: Jones and Bartlett Publishers, Inc.; 1999. p. 293-308.

3. Bienz K, Egger D, Pasamontes L. Association of polioviral proteins of the P2 genomic region with the viral replication complex and virus-induced membrane synthesis as visualized by electron microscopic immunocytochemistry and autoradiography. Virology. 1987;160(1):220-6. PubMed PMID: 2820130.

4. Melia CE, van der Schaar HM, Lyoo H, Limpens R, Feng Q, Wahedi M, et al. Escaping Host Factor PI4KB Inhibition: Enterovirus Genomic RNA Replication in the Absence of Replication Organelles. Cell reports. 2017;21(3):587-99. doi: 10.1016/j.celrep.2017.09.068. PubMed PMID: 29045829.

5. Melia CE, van der Schaar HM, de Jong AWM, Lyoo HR, Snijder EJ, Koster AJ, et al. The Origin, Dynamic Morphology, and PI4P-Independent Formation of Encephalomyocarditis Virus Replication Organelles. mBio. 2018;9(2). Epub 2018/04/19. doi: 10.1128/mBio.00420-18. PubMed PMID: 29666283; PubMed Central PMCID: PMCPMC5904412.
