# Supplementary material for: A unifying structural and functional model of the coronavirus replication organelle: Tracking down RNA synthesis
Source: PLoS Biol. 2020 Jun 8;18(6):e3000715. doi: 10.1371/journal.pbio.3000715 (PMC7302735; doi:10.1371/journal.pbio.3000715)
Supplement: Attachment — Submitted filename: ResponseToReviewers.docx [file pbio.3000715.s010.docx]

# Response to reviewer’s comments

**Several remarks from the reviewers raise similar points. Therefore, we will first address these jointly, grouped into two general points, before the detailed point-to-point response to each reviewer.**

## General points

1. It is not sufficiently clear what the different time points in infection used in this study represent relative to the virus replication cycle.

**We agree that this aspect was largely unclear in the original manuscript so we have tried to clarify this in the revised version (see changes below). It should be noted that our goal in this study was not to carry out a characterization of the coronavirus RO along infection, as this has been largely addressed before (see general point #2). In order to facilitate the EM (autoradiography) analysis, we chose representative time points close to the exponential phase of viral replication. When previously available (see general point #2), ultrastructural information was also considered to ensure a variety of abundant virus-induced membrane modification.**

**The selected time points using these criteria correspond to intermediate to late time points in infection, as can be assessed in the literature:**

- **MERS-CoV in Huh7 cells (12 hpi). Growth curve and vRNA synthesis in Fig. 1 in de Wilde et al. J. 2013. Gen. Virol. 94:1749.**
- **SARS-CoV in Vero E6 cells (7 & 9 hpi). Growth curve in Fig. 5 in Subissi et al. 2014. Proc Natl Acad Sci USA 111:E3900, vRNA synthesis in Fig. 1 in van der Worm et al. 2011. J. Virol. 85: 5669.**
- **MHV in 17Cl1 cells (8 hpi). Viral RNA synthesis in Fig. 1 in van der Worm et al. 2011. J. Virol. 85:5669.**
- **HCoV-229E in Huh7 cells (24 hpi). Growth curve in Fig. 1 in Muller et al. 2018. J. Virol. 92: e01463.**

**IBV infections in Vero cells are an exception to this general design. Due to the low titer of the virus stock and to maximize the number of positive cells, the time points (~16 hpi) were chosen to allow a second cycle of replication. Therefore, IBV-infected samples represent a more heterogenous set of infection stages.**

**In order to clarify this in the manuscript, we have explained our criteria for selecting time points in Material and Methods, introducing also the corresponding references (l. 564-569), and have made textual changes in the other parts of the manuscript (l. 111-115) and in the figure legends (l. 210, l. 256, l.364)**

2. The chronology of appearance of the different virus-induced membrane modifications is unclear.

**Indeed, we did not experimentally address this aspect in the current study as this has been analysed before for a few coronaviruses like SARS-CoV (Knops et al. 2008. PLoS Biol. 6:e226), MHV (Ulasli et al. 2010. Cell Microbiol. 12:844) and MERS-CoV (de Wilde et al. J. 2013. Gen. Virol. 94:1749). In all these studies it has become apparent that DMVs are the first virus-induced structures, while CM appear and proliferate later in infection. Clearly, this aspect should have been addressed in more detail to guide the reader. This is done now in l. 494-495 of the revised manuscript.**

Reviewer #1:

This manuscript by Snijder and co-workers entitled "A unifying structural and functional model of the coronavirus replication organelle: tracking down RNA synthesis" describes elegant studies aimed at discerning if double membrane vesicles or other ER sites modified by viral proteins are the site of coronavirus RNA synthesis during viral replication.  The authors describe that an early study using immuno-EM revealed that virus-induced double membrane vesicles (DMVs) were the sites of viral RNA synthesis (Gosert et al., 2002 Replication of mouse hepatitis virus takes place at double membrane vesicles, reference 14). With the advancements in EM techniques and particularly EM tomography, researchers appreciated that coronavirus replication induced reorganization of host membranes, generating both convoluted membranes (CM) and DMVs (Knoops et al., SARS-coronavirus replication is supported by a reticulovesicular network of modified ER, reference 15). In addition, the term double membrane spherules (DMS) was introduced to describe membrane rearrangements detected during the replication of infectious bronchitis virus (Maier et al., 2013 IBV generates spherules from zippered ER membranes, reference 29), but it was not clear if these structures were unique to avian coronaviruses, or if they played a role in RNA synthesis. In this study, the authors used radiolabeling of viral RNA and detected the labeled RNA in association with the DMVs. Importantly, they also determined that CMs, DMSs, and DMVs can be visualized for all the coronavirus specimens they studied, including MERS-CoV, suggesting that these structures are generated during the assembly of the viral replication complex and the DMVs necessary for viral RNA synthesis.

The technical work here is tremendous and the images of the viral protein-modified membranes are stunning.  However, the manuscript seems have an inordinate focus on the DMSs, potentially implicating these as unique (terminal?) entities. Isn't is possible that the DMS detected at the moment of fixation is in fact on its way to becoming a DMV? Alternatively, some of these DMSs could be unable to progress to DMV status, for reasons that are not yet clear. In the absence of live-cell EM, it is not possible to distinguish these possibilities, but certainly the possibility the DMS to DMV transition could be considered as the authors develop their "unifying structural and functional model" of coronavirus RNA synthesis.  Currently, there is a significant emphasis on the DMS in the manuscript, although this structure is not the take home message, and there is no functional role given to these structures, even as potential intermediates. If there is evidence that a DMS cannot
continue to grow into a DMV, then this should be made clear in the manuscript. Overall, the authors provide important results using state-of-the-art techniques that confirms and extends the early studies that that DMVs are the site of coronavirus RNA synthesis.

**In our view, the attention that DMSs receive in this manuscript matches the interest they attracted in the field when they were first described for IBV infection and proposed as sites for vRNA synthesis (see e.g. Neuman 2013. How the double spherules of infectious bronchitis virus impact our understanding of RNA virus replicative organelles.** **mBio 4:e00987). While our results do not support this possibility, this was a reasonable and attractive hypothesis given the architecture of DMSs and their similarity with the invaginations induced by multiple other +RNA viruses which are effectively used for vRNA synthesis .**

**The reviewer correctly points out that the possibility that DMS represent DMV precursors cannot be completely discarded. This may not be favoured by (1) the fact that there is a large gap between DMSs and DMVs and no intermediate structures were found, and (2) DMS seem to arise from CM, which are structures that are detected in infection later than DMVs (see general point #2). Still, it is entirely possible that CM and DMS may represent early stages in DMV formation that, for unclear reasons, are unable to progress to DMV at these point in infection. We acknowledge and discuss in more detail this hypothesis in (l. 489-501).**

Comments for the authors' consideration:
**1.**      Lines 375-378: Consider changing the order of this description to 1) CM and zippered membranes; ii) DMS and iii) DMVs.  This would allow you to speculate in a unifying model that coronaviruses modify membranes to promote "zippering", resulting in the formation of spherules that may mature into double membranes vesicles, the site of viral RNA synthesis.  This "progression" is still hypothetical because it has not be visualized in real time, but certainly all the data presented here are at least consistent with this unifying model.  One of the challenges is that researchers tend to see all of these structures simultaneously, and not a neat progression from one form to another. The discussion lines 410-413 seems to discount a progression model because of a lack of intermediate, but why couldn't a small double membrane spherule progress into a DMV? One could even speculate that the open DMS allows for capturing of a RNA that "seeds" the DMV to become a site of RNA synthesis.
It is reasonable to speculate about potential models in the discussion section, and to be open to multiple interpretations of the current findings.

**Following the reviewer’s recommendation we have changed the order in which the RO elements are presented (l. 439).**

**The possibility of DMS being precursors of DMVs is now discussed in more detail (see previous comment, changes in l. 489-501). Additionally, we have discussed in some more detail previous literature that sheds some light into the still unclear coronavirus DMV biogenesis. This indeed favours zippering as the first step in DMV biogenesis (l. 490-494)**

**2.**      Line 381: change "establish" to confirms and extends. This manuscript works very hard at presenting uncertainty or controversy about coronavirus replication sites.  However, the questions that arose about the sites of RNA synthesis came about as the techniques to visualize the modified membranes were improved over time, suggesting the potential for more than the originally documented DMVs were involved RNA synthesis. Perhaps this is a stylistic point, but pointing out that the results here confirm and extend earlier studies is important and accurate.

**This is fair point so the suggested change has been implemented (l. 445)**

**3.**      The text could be much more concise by focusing on the idea that this work is evaluating all structures in multiple coronaviruses, which has not been done before in one manuscript, and seeking to determine if one or multiple sites are used for viral RNA synthesis.  The "controversy" seems vastly over emphasized here.

**The other reviewers did not request shortening the manuscript, seemed to enjoy reading it in its current form (e.g. reviewer #2: “I found the manuscript fun to read with beautiful images”), and acknowledged the confusing situation regarding the central question addressed in this article (reviewer #3: “The main point of the present study is the clarification of a long-standing debate by showing that DMVs most likely are the site of viral replication”). Therefore, and although we have tried to introduce some changes in the indicated direction, we believe that this may be largely a matter of opinion. If the reviewer thinks this is very important, we could certainly reconsider this point, but in that case it would be good to receive more specific indications as to which parts of the text should be shortened and/or rephrased to avoid ‘overemphasizing the controversy’. We believe we mainly indicated that ‘the picture was unclear’ so far, and has been clarified in this study.**

**4.**      The images in the supplementary section are striking also.  If PLoS Biology allows it, try to include all figures in the body of the paper and have only the movie in the supplement.

**We thank the reviewer for the enthusiasm and appreciation of the images that we present here. Since there is no limit in PLoS Biology for the number of figures, and following the reviewer’s recommendation, we propose in this resubmission to include former S1 Fig and S3 Fig in the body of the paper, as Fig 2 and Fig 7, respectively. Since former figure S2 Fig refers to previous studies, it feels more appropriate to keep it as Supporting Information (new S1 Fig)**

Reviewer #2:

Coronaviruses have emerged as important zoonotic viruses with major threat to human health. In this paper, the authors characterized the ultra structures of human cells infected by different coronaviruses including MERS, SARS-CoV1, MHV, HCoV-229E and IBV. They have identified three different membranous structures, DMVs, CM and DMS, which have all been observed before in various coronavirus infections. The detection of DMS is new for MERS infection. Importantly, they were able now to provide a more universal picture that all these viruses induce the formation of all these RO structures. By using metabolic labeling of viral RNAs, after shutting down cellular transcription, they were able to demonstrate that DMVs are the likely places for coronavirus RNA synthesis and replication. Somewhat surprisingly, they find no significant roles for CM and DMS in coronavirus RNA synthesis and replication. This conclusion was further supported by detection of viral dsRNAs within DMVs.

The findings are potentially important, especially the metabolic labeling data are quite exciting. I found the manuscript is fun to read with beautiful images. The manuscript requires the following changes.

Fig. 1 shows images after 12 h incubation with many DMSs. What time point are these DMSs first appear?

**While we don’t know exactly the first time point in which DMSs arise, the order of appearance of MERS-CoV-induced structures along infection is clear. Our previous analysis of MERS-CoV-infected samples, (de Wilde et al. J. 2013. Gen. Virol. 94:1749, re-examined in this study in S1 Fig -formerly, S2 Fig), clearly indicated that both DMSs and CM (from which DMSs seem to arise) appear later in infection than DMVs. The concern of the reviewer seems to be related to the general points #1 and #2 addressed at the beginning of this rebuttal. Hopefully, the changes indicated there clarify this matter.**

Fig. 1 and Fig. 6. Please replace the arrowheads with different colors to aid the readers. Gray and black arrowheads could be quite similar on different computer screens.

**This is an very good suggestion that we have now implemented for Fig. 1. To keep former Fig 6 (now Fig. 8) as a greyscale figure, we have changed the grey arrowheads into black arrows.**

Fig. 1F:  please define what are arrows pointing at?

**We thank the reviewer for spotting this omission. The arrows point at constrictions in the DMVs, which are now described in the text (l. 181-184) and indicated in the figure legend l. 151)**

Fig. 1F: Are the outer DMVs and the ER membranes are continuous membranes or are there gaps between them. It seems that they are continuous based on the presented images here.

**This is indeed the case. DMV-ER connections are indicated with white arrowheads in Fig. 1F, and mentioned in the figure legend and in the text (l. 187, now making explicit that the connection is established through DMV outer membrane)**

Fig. 4B:  it would be useful to pinpoint DMVs, since it is very crowded RO is shown here.

**Recommendation followed (see new Fig 5, yellow asterisks)**

Fig. 5. It would be useful to know what % of label was inside versus outside of DMVs? It looks more label is on the outside of DMVs (although very nearby to the DMVs). What is the possible explanation?

**We believe that the reviewer refers to former Fig. 4 (Fig 5 in the new submission), as the point is about percentages of label inside/outside DMVs. The reviewer’s observation is correct: there is more signal outside than inside the DMVs: 75% outside, 9% inside DMVs and 25% on the DMV membranes. (The numbers can now be calculated by any interested reader from the additional S4 Data file, that contains all the numerical data associated with the figure). However, this is a logical result considering the characteristics of autoradiography, and should not be overinterpreted (see l. 270-272 in the manuscript, S1 Text). The electrons emitted after a radioactive disintegration can travel in any direction before hitting the photographic emulsion at the location where, ultimately, the autoradiography grain will be visible. Accordingly, autoradiography signal follows a probability distribution around the radioactive source that can extend several hundred nanometers away from it. In comparison, DMVs are relatively small structures (~250 nm in diameter), so a labelled newly-synthesized vRNA copy putatively produced inside a DMV (e.g. in the inner membrane) is more likely to generate signal outside than inside the DMV.**

**Additionally, it is worth considering that the perimeter of the scanned area increases with the distance to the DMV centre, which results in higher counts of background autoradiography grains as more distant areas are sampled. Finally, an additional factor that could contribute to the higher number of counts outside the DMVs is the migration of a fraction of the newly-synthesized vRNA from the DMVs.**

**In summary, while the signal distribution around DMVs demonstrates that newly-synthesized vRNA is associated with DMVs, the higher percentage of signal outside the DMVs is to be expected regardless of the exact location of vRNA synthesis. Unfortunately, the signal distribution does not provide additional clues on this matter.**

Lane 155:  based on the findings in this work, I doubt it is correct to call zippered ER and CM as ROs, since no viral RNAs were detected in those structures.

**This is certainly a valid point. We have modified the corresponding text accordingly (l. 178).**

L407:   CM and DMS biogenesis does look very abundant at 12 time point in Fig. 1A-B, contradicting that these structures form late during infection. What is the first time point when CM and DMS structures are detected? The time of the vents for the biogenesis of various RO structures is confusing in this work.

**Although 12hpi can indeed be considered a relatively advanced point in infection (Fig. 1 in de Wilde et al. J. 2013. Gen. Virol. 94: 1749, it would have been more appropriate to state that CM and DMS appear later in infection than DMVs, and therefore the text has been adjusted accordingly (l. 494-495). The question is essentially the same as that formulated in the first point by this reviewer. Also, it relates to the general points 1 & 2 discussed at the beginning of this review, which we have addressed in much more detail in the revised manuscript.**

Please discuss the origin of DMVs, since the ER origin of CM and DMS is discussed nicely in the discussion part.

**This is now included in the discussion (l. 491-494), also considering the comments of reviewer 1.**

I note that DMSs are possibly too small to harbor the large coronavirus RNAs, possibly dsRNA (if that is the template for RNA synthesis), sgRNAs and the replicase complex, while copying the RNA templates. Even the 6x times smaller tombusviruses and nodaviruses form almost that size spherules to support replication as the observed size of DMS in this work.
Is it possible that DMSs are high-density membrane-protein (both viral and host-derived) storage granules to reduce ER stress in the cells?

**The remarkable large size of the coronavirus genomic RNA (26-34 kb) could indeed create constraints for the use of DMSs as efficient sites of vRNA synthesis. Although we are not aware of any DMS-equivalent structure in the context of ER stress, this may be an interesting possibility to explore. This and other possible DMS roles certainly warrant future investigation.**

Do the authors have data on how much percentage of the label is incorporated into genomic versus subgenomic RNA species during metabolic labeling? That would be useful information.

**Different studies have shown that genomic viral RNAs represents only a small percentage: 4% in MERS-CoV-infected cells, 5% in SARS-CoV infection, 6% in SARS-CoV-2 infection (de Wilde et al. J. 2013. Gen. Virol. 94: 1749, Ogando et al. https://doi.org/10.1101/2020.04.20.049924 ). The relative abundance of the genomic and subgenomic RNAs produced in MERS-CoV-infected cells (Fig 1b, de Wilde et al. 2013) can be translated into percentages of incorporated label in our assays after compensating for the differences in size and uracil content of each of these different viral RNAs. In this way, we calculated that ~32% of the radioactive label would be incorporated into genomic RNA vs 68% into subgenomic RNAs. These estimates have been now incorporated in the text (l. 220-227)**

Reviewer #3:

In this manuscript, the authors performed a detailed structural and functional characterization of the replication organelles (ROs) of coronaviruses. Coronavirus ROs are composed of predominantly double membrane vesicles (DMVs). Through comparative analysis, the authors demonstrate that convoluted membranes, zippered ER and double-membrane spherules are conserved across alpha-, beta- and gamma- CoVs and altogether form the viral ROs. Additionally, the authors employed a classical, yet elegant approach that combines ultrastructural analysis with RNA labeling using radiolabeled nucleosides to demonstrate that viral RNA synthesis is associated with double membrane vesicles. This finding provides best available evidence so far that DMVs are the site of viral RNA replication.

This is a timely study that sheds light on the role of the different membranous structures observed during CoV replication, although these structures have been described in several earlier reports. The main point of the present study is the clarification of a long-standing debate by showing that DMVs most likely are the site of viral RNA replication.

Major comment:
The results of this study are clear and the author´s claims are supported by their data. One of the aims was to clarify the apparent differences reported in the ultrastructural architecture of the coronaviral ROs. The authors achieved their goal by showing that the ROs of the different CoVs are essentially the same.  One of the limitations is that the authors provide a single snapshot looking at a single time point for the different viruses, often in different cells and using different MOIs. Moreover, the authors do not provide a growth curve of the viruses in the different cell lines. This information would be useful to allow for a direct comparison between the different conditions. The authors could at least comment if the selected time points reflect the initial phase of the viral infection or a later phase.

**The reviewer here raises a point that was also mentioned by the other reviewers, clearly showing that this aspect needed to be better addressed in the manuscript. We have responded to these concerns jointly at the beginning of the rebuttal (see general points 1 & 2, and changes in the text indicated therein). As explained there, and now also in the manuscript, the selected time points represent intermediate-late infection stages. This information is now present in the text. Appropriate references to evaluate this (e.g. through growth curves), have also been included in the manuscript (l. 565).**

**Due to differences in receptor usage, resorting to different (susceptible) cell lines is often unavoidable to study infection with different coronaviruses. While it is true that this diversity makes comparisons more difficult, we also believe that fact that similar results were obtained in these different conditions further support our conclusions.**

Additionally, what is the order of appearance of the different structures forming the CoV ROs and is this order conserved among the different CoVs? This could support the idea of a general mechanism used by all CoVs and the regulation of the biogenesis of their ROs. If such information is already present in the literature the author should comment on this point in the discussion.

**Please, see general point 2. As similar points were raised by reviewers 1 & 2, we opted for a joint response at the beginning of this rebuttal. The biogenesis of the different RO elements is also now addressed in more detail in the discussion (l. 489-501)**

Minor points:
For the autoradiography experiments time points, MOIs and cell types differ between MERS-CoV and SARS-CoV infections. Are the time points selected by the authors comparable in terms of progression of viral infection of the two viruses in the different experimental conditions? In other words, are the selected time points within the exponential phase of viral replication or at the plateau phase of viral replication? The authors should comment on this.

**This again relates to the general point 1 (please, see also there for specific changes implemented in the manuscript). The time points for autoradiography selected for SARS-CoV in Vero E6 cells and MERS-CoV in Huh7 cells are both within the advanced phase of exponential viral replication (see van der Worm et al. 2011. J. Virol. 85: 5669, and de Wilde et al. J. 2013. Gen. Virol. 94: 1749), although SARS-CoV infection likely represents a slightly earlier stage than MERS-CoV infection. It should be noted that ultrastructural considerations based on our previous studies (Knops et al. 2008. PLoS Biol. 6: e226, de Wilde et al. J. 2013. Gen. Virol. 94: 1749), were also a factor that guided the selection of time points, as our aim was to obtain samples with an abundant representation of the different RO elements under analysis.**

**4.** Line 413 - there is no reference to backup this claim. Since this study does not analyze how the RO architecture changes during infection, the claim that DMV formation precedes the appearance of CMs should come from a previous study. The authors should add the reference of the paper to which they are referring.

**This was indeed an important detail missing. The pre-existing knowledge about the order of appearance of the different virus-induced structure is now addressed in more detail in the manuscript (l. 494-495, see also general point 2).**

**5.** FigS2- the figure legend states that Huh7 cells were infected with MERS-CoV while the figure annotation says Vero cells. The authors should clarify.

**These were Vero cells. We thank the reviewer for spotting this mistake, which has now been mended in the figure legend (new S1 Fig, see l. 1065).**

6. Line 177 - truncated sentence

**Again, we thank the reviewer for pointing out this mistake. Corrected in l. 205.**

## Note for the editor

**Following the editor’s recommendations, we have submitted a step-by-step protocol for the preparation of autoradiography samples in protocols.io (**<http://dx.doi.org/10.17504/protocols.io.bfrtjm6n>)**. Data files with the underlying numerical data supporting the results are now also included.**
